# ENIGMA-Viewer: interactive visualization strategies for conveying effect sizes in meta-analysis

**DOI:** 10.1186/s12859-017-1634-8

**Published:** 2017-06-06

**Authors:** Guohao Zhang, Peter Kochunov, Elliot Hong, Sinead Kelly, Christopher Whelan, Neda Jahanshad, Paul Thompson, Jian Chen

**Affiliations:** 1 Department of Computer Science and Electrical Engineering, University of Maryland, Baltimore County, 1000 Hilltop Circle, Baltimore, 21250 MD USA; 2 Maryland Psychiatric Research Center, University of Maryland, Baltimore, 55 Wade Ave, Baltimore, 21228 MD USA; 3 Keck School of Medicine, University of Southern California, 1975 Zonal Ave, Los Angeles, 90033 LA USA; 4 Department of Psychiatry, Beth Israel Deaconess Medical Center, Harvard Medical School, 330 Brookline Ave, Boston, 02215 MA USA; 5 Psychiatry Neuroimaging Laboratory, Brigham and Women’s Hospital, Harvard Medical School, 75 Francis St, Boston, 02115 MA USA; 6 Imaging Genetics Center, Mark and Mary Stevens Neuroimaging and Informatics Institute, University of Southern California, 1975 Zonal Ave, Los Angeles, 90033 LA USA; 7 Department of Molecular and Cellular Therapeutics, Royal College of Surgeons in Ireland, Dublin, 123 St Stephen’s Green, Dublin 2, Ireland

**Keywords:** Interactive visualization, Meta-analysis, Effect size, Diffusion tensor imaging, Comparative studies

## Abstract

**Background:**

Global scale brain research collaborations such as the ENIGMA (Enhancing Neuro Imaging Genetics through Meta Analysis) consortium are beginning to collect data in large quantity and to conduct meta-analyses using uniformed protocols. It becomes strategically important that the results can be communicated among brain scientists effectively. Traditional graphs and charts failed to convey the complex shapes of brain structures which are essential to the understanding of the result statistics from the analyses. These problems could be addressed using interactive visualization strategies that can link those statistics with brain structures in order to provide a better interface to understand brain research results.

**Results:**

We present ENIGMA-Viewer, an interactive web-based visualization tool for brain scientists to compare statistics such as effect sizes from meta-analysis results on standardized ROIs (regions-of-interest) across multiple studies. The tool incorporates visualization design principles such as focus+context and visual data fusion to enable users to better understand the statistics on brain structures. To demonstrate the usability of the tool, three examples using recent research data are discussed via case studies.

**Conclusions:**

ENIGMA-Viewer supports presentations and communications of brain research results through effective visualization designs. By linking visualizations of both statistics and structures, users can gain more insights into the presented data that are otherwise difficult to obtain. ENIGMA-Viewer is an open-source tool, the source code and sample data are publicly accessible through the NITRC website (http://www.nitrc.org/projects/enigmaviewer_20). The tool can also be directly accessed online (http://enigma-viewer.org).

## Background

Large scale harmonization of image processing protocols across different studies around the world and the extraction of effect sizes across reliably extracted regions of interest, allows for a common framework though which results can be compared, and combined through unbiased meta-analyses as performed in the ENIGMA (Enhancing Neuro Imaging Genetics through Meta-Analysis) consortium [1]. These advancements offer essential opportunities for brain scientists to produce credible findings through meta-analysis, a method that combines data cohorts collected worldwide to obtain the statistical power otherwise unavailable from a single cohort, in order to find cross-modality data associations that influence brain structures [2]. Cohort studies boost power to detect associations. Seminal accomplishments with promising results in imaging-genomics associations have accelerated scientific discoveries in areas such as schizophrenia [3], bipolar disorders [4], and other neurodegenerative diseases [5].

In meta-analysis, one important task is to interpret effect size, a statistical measure that can be broadly defined as any statistic that quantifies the degree to which sample results diverge from the expectations in the null hypothesis. Computing effect size is important because if effect sizes are stable across studies or even generalizable over some variations in design or analysis, the results are replicable. That is, effect size is a statistical tool for meta-analysis that quantitatively synthesizes effects across different studies. Ranking brain measures in order of their effect sizes for case-control differences can unearth brain measures on the basis of both the stability of the brain volume measures (so-called heritability [6]) and their relevance in the disease being studied [7].

Comparing effect sizes is, however, a multi-variant issue, not only because scientists must choose studies carefully to ensure consistency of protocol use, but also because the variety of cohorts has made it possible to dig more deeply into and disentangle the sources (medication-related geographical or demographics and genetic factors, e.g. [8]) of variations that could explain why brain differences vary across studies and different phenotypes.

As new analytical results are produced and lead to increased data dimensionality and size, the bottleneck to human understanding is not only limited to data mining and computational approaches, but also to human limited memory capacity. Presenting and interpreting effect sizes and locating regions across studies can be obscured due to the complexity of interpreting the multivariate information space and the problems inherent in presenting rich datasets on a two-dimensional (2D) computer screen. Synthesizing new information for new discoveries and comparison with past results is cognitively demanding. The bandwidth of discovery will be bounded by the characteristics of human perception, and hence the quest for visualization has commenced in the brain sciences, as evidenced by recent reviews and by research on the vital role of visualization in the analysis of multimodal neuroimaging data [9–11].

**Fig. 1:**
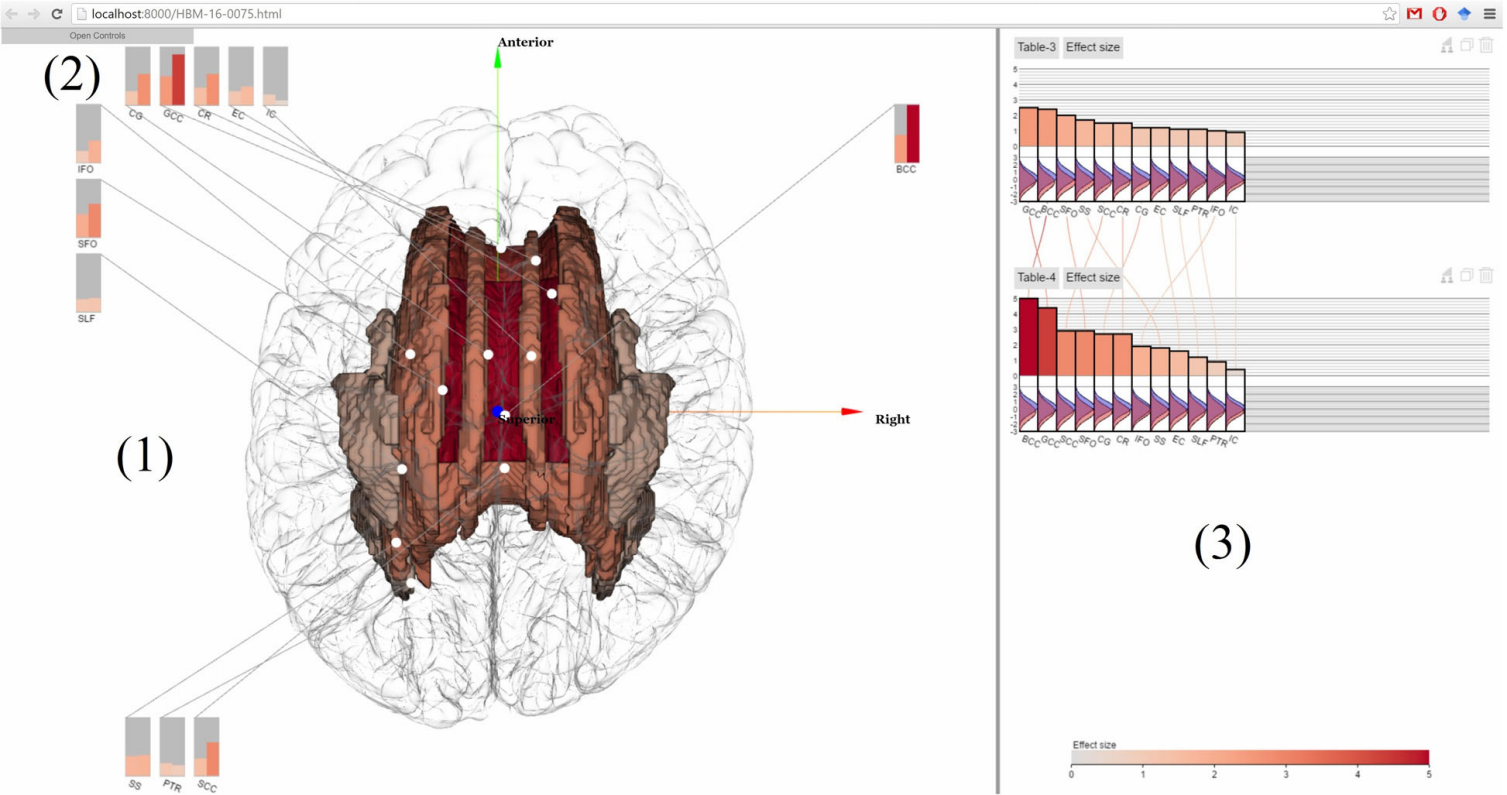
ENIGMA-Viewer Interface. Data are taken from a comparison of normal control and a diseased brain in [33]. (*1*) Region-centric view. Brain white matter regions of interest from the ENIGMA-DTI protocols are shown inside a glass brain. The colors encode the effect sizes using the same color scale as that used in the bar chart. The in-place bar charts (*2*) are also drawn to facility comparison of regions across multiple studies. (*3*) Study-centric view. Both the bar height and bar color represent effect sizes and the bars are sorted from largest to smallest within each study. This example shows the patient-control comparison statistics: effect sizes for fractional anisotropy (FA). Distributions of per-subject FA values for patient (*red*) and control (*blue*) group are illustrated as curves beneath the bars of each of these brain regions
- Consideration of the brain science domain and tools to aid multivariate comparison studies.
- Priority in integrating different imaging modalities to compare results and locate important information.

Our long-term goals include making analytics results derived from the ENIGMA pipeline accessible to the neuroscience community at large and assisting brain scientists in seeing patterns in massive multimodal computational solutions, as well as encouraging effective communication and collaborative activities through visual means to convey our results to the general public. Here we present ENIGMA-Viewer (Fig. 1), an interactive visualization tool to let users explore multimodality brain data to compare effect sizes and associated brain anatomical structures and genomics factors. This work makes several contributions:

- A series of design strategies for spatial and non-spatial data integration in the context of meta-analysis of brain imaging and genetics.

## Related work

Both neuroscience and visualization scientists have worked extensively on visualizing brain datasets. This section reviews related work in visualization, related data analysis, and multi-modality data visualization.

### Brain data visualization

Many brain data visualization tools have addressed important issues in conveying single modality imaging techniques. In diffusion tensor magnetic resonance imaging (DTI) data visualization, Laidlaw et al. designed multivariate tensor field visualization at every voxel using creative artistic rendering [12]. Other powerful techniques have used tensor glyphs to convey tensor shapes [13], or non-photorealistic rendering to resolve complex spatial depth perception [14] as well as validating studies in the large display uses [15] and rendering solutions [16, 17]. Functional brain network (fMRI) visualizations have showed bundling 3D trajectories can support functional network understanding in both 3D [18] or 2D connectivity studies in matrix views [19].

Despite these creative solutions and technical advances, none of the work to our knowledge has exploited features and interpreting results across multiple modalities and multiple datasets, except our own work by Novak et al. [10] and Zhang et al. [20]. A main difference between single and cohort analyses is that single images become unimportant and statistical results comparing cohorts can lead to valuable understanding of associations between brain regions and diseases. Kehrer et al. have laid out important design challenges in multimodality multi-faceted data visualization in the broad medical imaging areas related to comparative studies as well as possible solutions in the use of multiview visualization to represent multidimensional data [21]. Our current work follows the multiview solutions to let scientists visually synthesize results from different views.

Other work most closely related to ours is Novak et al.’s EnigmaVis. That work lets scientists make quick comparisons among new and existing DTI-GWAS (genome-wide association study) queries through a powerful web interface [10]. This pioneering study is significant because it supports quick hypothesis confirmation through comparisons and lets brain scientists explore studies and examines results before their own study is conducted. However, that tool generates fixed images and only supports limited interactivity. Our design advances visual exploration by supporting interactive data exploration especially not only for presenting and combining different imaging and measurements results but also for comparative visualization between modalities. Our design is very different from that of EnigmaVis in that no prior hypotheses or knowledge of prior studies is required to explore the prior studies in an interactive environment. Brain scientists can load and compare their data. We believe our solution can have great potential to support opportunistic discovery and may enable scientists to more easily and interactively investigate broader scientific questions.

### Integrating spatial and non-spatial data

Our solution to comparative effect sizes is related to spatial and non-spatial data integration to assist data analysis. Our choices of visualization is mostly driven by data types, which is similar to the design rationale in Keefe et al. [22], where they visualize quantitative parameters using non-spatial data visualization to avoid inaccurate judgment of three-dimensional measurement. Wang and Tao also defines the integration of spatial and non-spatial data visualization [23], as well as Chen, Pyla, and Bowman in three-dimensional interface design [24].

## Scientific background and data source

This section describes the background that motivates our visualization design, followed by description of the data used in the visualization.

### Introduction to the goals in ENIGMA DTI-GWAS data analysis

The ENIGMA consortium aims to enable image-genetics discoveries by examining reproducibility, heritability, and association with diseases through analyzing brain imaging measures and genotypes [1]. The goal is to address the most fundamental questions in neuroscience by linking brain brain measures to human well being. Some of the most intriguing questions include: *what are the effects of aging, degenerative disease and psychiatric illness on the living brain?* *How do brain measures relate to cognition and behavior?* *Do brain measures predict our risk for disease, or give prognoses for those who are ill?* [1]

The method is meta-analysis, a quantitative statistical analysis of several separate but similar experiments or studies using pre-agreed covariates in order to test the pooled data and examine the effectiveness of the results [1]. Subsequently, the *p*-values and regression coefficients are combined by weighting the results based on the sample size of each contributing cohort. Meta-analysis is not only important for brain white-matter analysis, but it has been the only way to find credible genetic traits of brain disorders with sufficient statistical power to achieve significant effects greater than *p*<10^−8^.

Great advancements in related fields have laid foundations for making cohort-comparison possible by addressing challenging technical problems in multiple areas. These include creating common ENIGMA template [2], harmonization of protocols to synthesize data captured with different protocols [25], generating tract-based spatial statistics skeletonization [26], regions of interests (ROI) extraction [27], and SOLAR statistics [28]. Meta-analyses have also identified the stabilities of brain volume measures (or heritability) in sub-cortical (containing regions associated with human function) and cortical regions across twins, genders, and geolocations [29].

A common workflow in performing meta-analysis is first to follow pre-determined protocols to obtain desirable imaging modalities (here DTI) and genomics in the population under investigation. Tract or voxel-based analyses and associated metrics measures (e.g., fractional anisotropy (FA) or water diffusion and cortical thickness) sensitive to the neuro-degenerations are then derived. Effect sizes in DTI studies are quantitatively compared.

### Data

**Brain imaging data** The 3D brain imaging dataset labeled total 48 white matter structures in the JHU white matter atlas [30]. The brain volume in this atlas has 182×218×182 voxels measured at the resolution of 1×1×1 millimeters. We extract the surface mesh for each white matter region from the atlas using marching-cubes [31]. For cortical regions, we use cortical meshes from FreeSurfer. Since the FreeSurfer and the JHU atlases are different, the 70 FreeSurfer cortical regions are transformed to by matching the atlases using the linear transformation function in FSL [32].

**Statistical analysis data** The statistical data used in the program are from recent studies from ENIGMA group. For these analyses, effect sizes are reported as overall Cohen’s d values for case/control effects and some studies also report Z-scores for quantitative effects (such as FA values for white matter studies) from linear regressions of individual subjects. An example data is from the study on the heterochronicity of white matter development and effect of aging in schizophrenia [33]. That study computes effect size values for 12 affected brain white matter regions contained in the JHU atalas.

## Task analysis

The first goal of this study is to characterize the problems being addressed by the brain scientists as visualization tasks.

### Procedure

The task analysis was achieved by working closely with brain scientists, as well as by literature review. Each scientist was interviewed to gather sufficient information on their workflow tasks and goals. Each participant also used our prototype tool of ENIGMA-Viewer and suggested action steps and desirable outcomes. To collect the resulting feedback, we have asked them to answer the following questions: What kinds of questions do you anticipate exploring using the visualization tool? What would you like to achieve using visualization in general, communication or seeing patterns? Why do the state-of-the-art tools, such as AFNI [34], FSL [35], DtiStudio [36], not address your needs? How would you like the data to be depicted and represented? Should the data be visualized in 2D or 3D? How would you like to interact with and explore the datasets?

### Task list

Neuroscientists are interested in detecting trends and viewing overall data distribution as well as individual regions of interest. The most important tasks are related to (1) comparing similarities and differences in different disorders or in disease and control conditions; (2) comparing effect sizes in meta-analysis to find the truly significant brain regions and associated genetics factors; (3) studying the most important genetics association with these brain regions to establish the DTI-GWAS association; (4) identifying brain regions with high and low heritability. Each of these domain tasks can be abstracted to the fundamental analytic tasks presented by Amar, Eagan, and Stasko [37] and Schulz et al. [38], as listed in Table 1.

**Table 1:** DTI-GWAS Task List. Our ENIGMA-Viewer is designed for these analytical tasks

Index	Task	Example questions
1	Viewing distribution,	What is the most affected brain region under a certain disorder?
	consistency, or	What is the overall data distribution of the effect size in all studies?
	inconsistency	Are the results consistent or very different?
2	Detect trends	What is the distribution of cortical thicknesses and FA values?
		How does effect size vary among studies?
3	Find association	What are the disorder (brain regions) and genetics correlates to risk?
		How does brain structural change associated with
		behavioral risks and changes and vulnerability?
		How do geographical factors affect disease expression in the brain?
4	Locate extremes	What are the significant brain regions mostly affected by diseases?
5	Find local relationships	What are the differences between studies in terms of their effect size?
6	Compare different disorders	What are the common and different effects in the brain networks
		between or among multiple disorders?
7	Compare disorders by regions	What are the regions affected or unique to a certain disorder?

## Methods

This section presents our main contribution, i.e., the design decisions made in the ENIGMA-Viewer to address all those users’ tasks.

### Overview of the design considerations

The possibilities for encoding and interacting with the data mentioned in Related work section are vast. Our encodings and layout draw upon existing idioms, and our task framework suggests that more novelty is required. We investigate visual design options through our experience of working on interface layout, discussion among the team of co-authors and following good design principles. We have also designed interaction techniques so that results from one data type and modality can guide comparative analysis of another in a unified interface level. Data belonging to different types can be visually linked through interaction.

We use *juxtaposition*, which places effect sizes, 3D anatomical regions, and artificial GWAS side-by-side in small-multiples displays similar to that of Chen et al. [39]. We also use *superposition* which the effect sizes and 3D anatomical regions are overlaid in the same frame of reference, following the comparative visualization classification by Gleicher et al. [40] and Karnick et al. [41].

### Visual data fusion

Visual data fusion intermixes different facets of scientific data in a single view using a common frame of reference. In our program, effect sizes in different study cohorts and 3D anatomical regions can be grouped and presented in the space of the 3D glass brain. This visualization addresses scenarios of use in which a brain scientist wants to focus on finding associations of effect sizes in one or more regions of interests. For example, a brain scientist can load new and existing studies and then inspect trends and differences among studies visually. Another example use is to study multiple closely proximate brain regions of cortical and sub-cortical regions. Data from these spatial locations and multiple effect sizes can be discussed together. When the brain scientists’ task is to search for associative relationships between different studies in a common region of interest, this visual fusion would be appropriate to let the user focus simply on one view to obtain all information.

### Focus+context

Focus+context visualization supports both focused and detailed views as well as context for navigation purpose. Effect sizes of each cohort are displayed in small multiples using bar charts ordered by effect size magnitudes. Since the effect sizes vary across studies, using uniform-scale bar charts would render smaller effect sizes too small to be distinguishable visually. Our solution is to color the magnitudes of the effect sizes. This strategy introduces dual encoding to encode the magnitudes of effect sizes: the bars use length with the most precise magnitude discrimination, while colors encourage pattern finding to locate extreme effect size magnitudes in different cohorts. The diverging color map is perceptually linear and the zero mark appears where the two colors intersect at 0 to represent the least significant effect size. Positive effect sizes are mapped to red and negative effect sizes are mapped to blue. In this way, users can obtain at a glance the most significant brain regions by searching for the most saturated red or blue regions. We plot the FA distributions between the patient and control cohorts in order to show the FA differences. Here we can observe that the control cohort has higher average FA values then the diseased ones in all regions.

### Reduce context switching cost

**Fig. 2:**
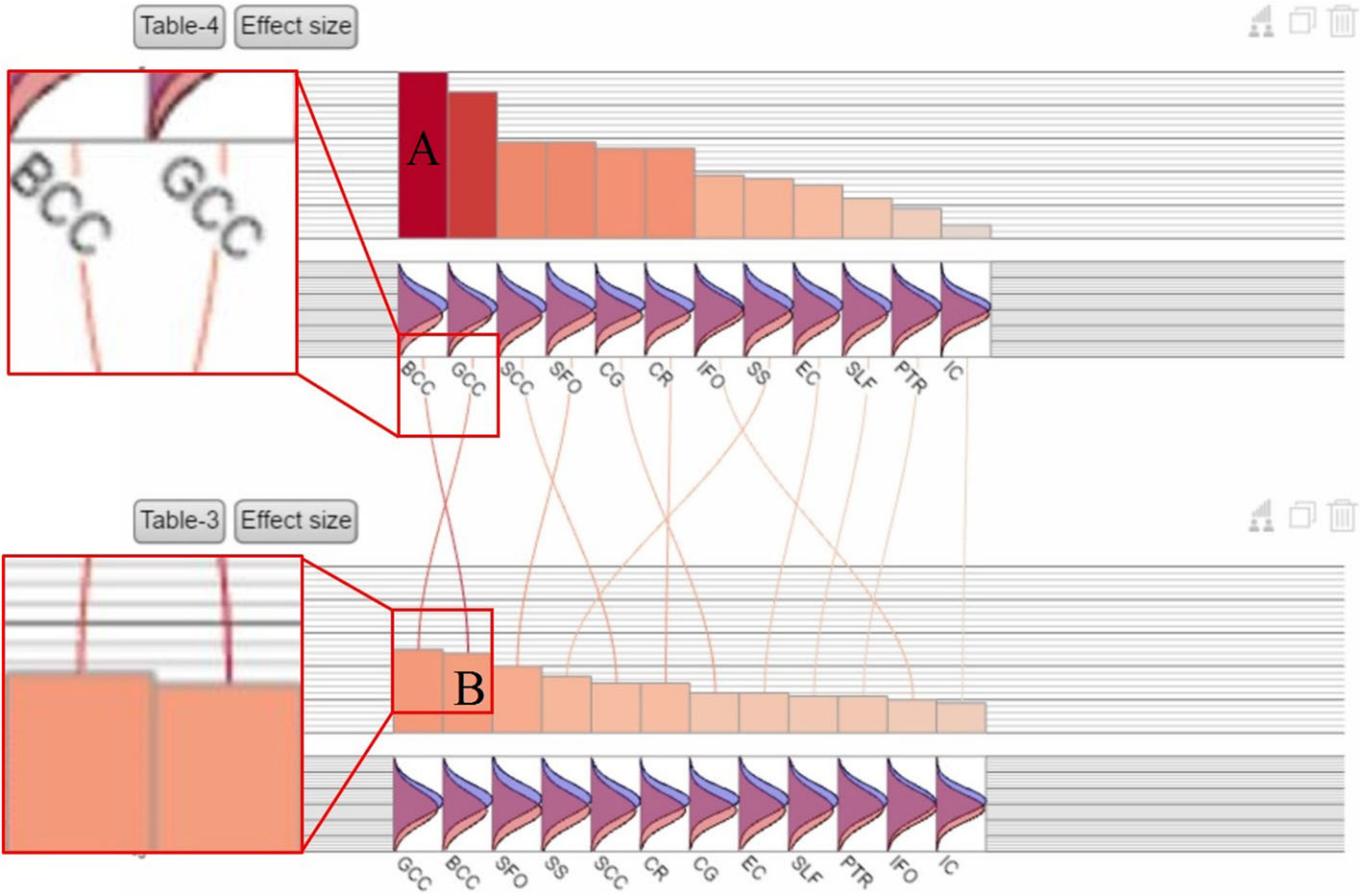
Scented edges to reduce the context switching cost. Here *curved lines* connect the corresponding regions between two studies (*top* and *bottom* ones). The color on each curve varies gradually in the way that the color in the top bar uses the color in the bottom one and vice versa. In this way, a viewer does not need to trace the link to compare study differences

The cost of context switching in visualization [42, 43] is one drawback of the small-multiples display in which bar charts are placed side- by-side. For searching for association between studies, the viewer must constantly switch the viewpoint between studies to look for relevant information in other views. To reduce the cost, our current method is to use “information scent” [44, 45], nuances added to the display to help the user construct visual associations. The edges are “scented” using the color representing the effect size magnitude in the neighboring view so that the user need not visually trace the edge to learn the magnitude in the other view (Fig. 2).

**Fig. 3:**
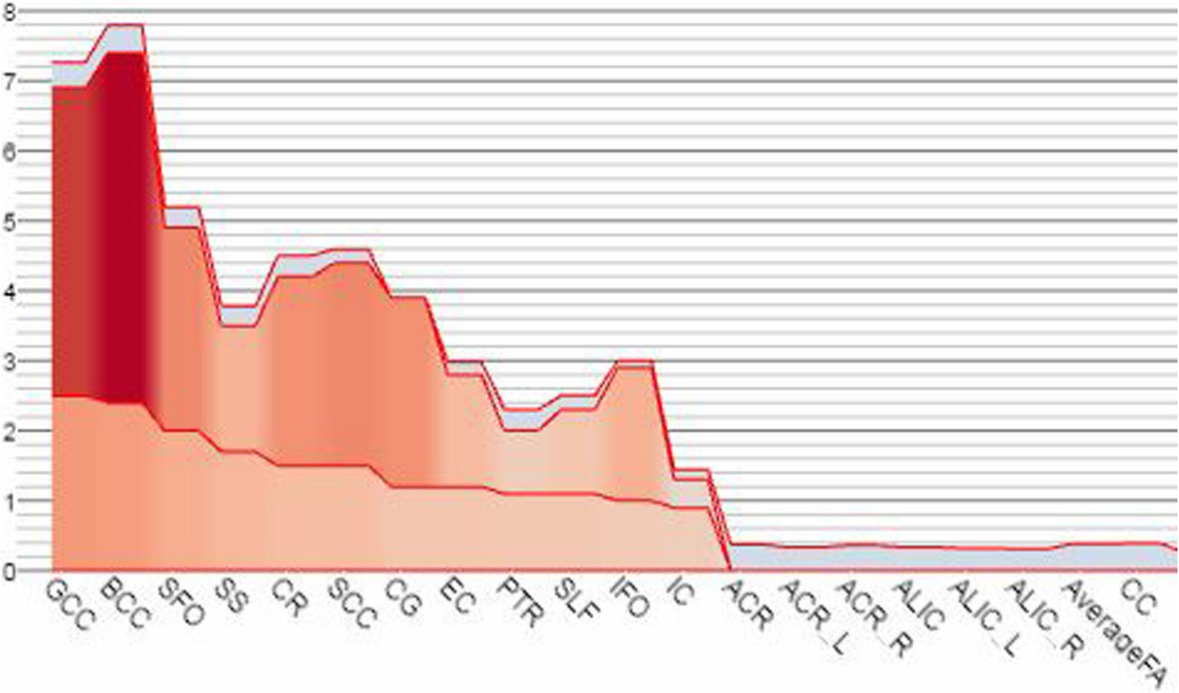
Effect sizes are stacked together to support comparisons among cohorts in different brain regions. Horizontally, the cohorts are ordered by the effect size magnitudes in the bottom cohort. A viewer can easily finds differences between studies

The second way to reduce the context switching cost and to facilitate comparison of common regions is to use the stacked bar chart (Fig. 3). Effect sizes in the same brain region belonging to different cohorts are stacked together and horizontally, the cohorts are ordered by the effect size magnitudes in the bottom cohort. This view facilitates both between and within effect sizes of the same and different brain regions and saves space. It is also easy to find region choice discrepancies between or among studies because some studies many include more regions than others.

**Fig. 4:**
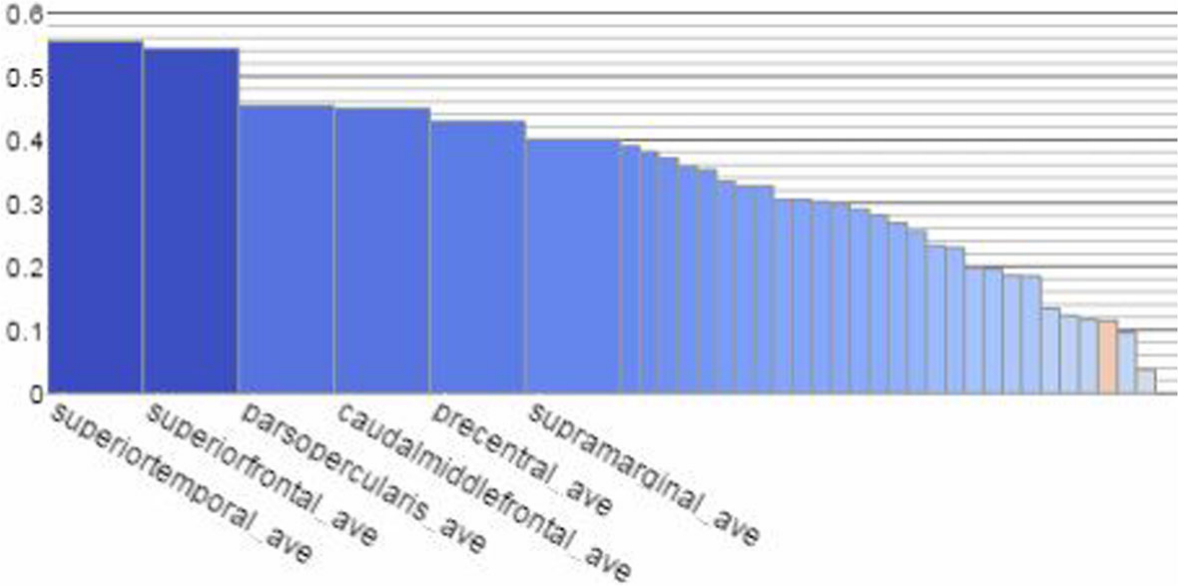
Bars can be re-scaled to make whole dataset visible in one view while leaving regions with large effect size enough screen space. In this example, bars with effect size smaller than 0.4 have their width narrowed to one fifth of normal width

Our design follows importance-driven interactions. If the screen space is not enough to show all bars for all regions, we keep the important ones, e.g. those with large effect size, unchanged and make less important ones smaller and in context. This scaling mechanism makes the larger effect size regions visually salient. The user can directly interact with the views to rescale the size of the bar charts. Figure 4 shows an example where the bars with the effect sizes lower than 0.4 are toggled to have one fifth of normal bar width and their labels hidden.

### View reconfiguration

**Fig. 5:**
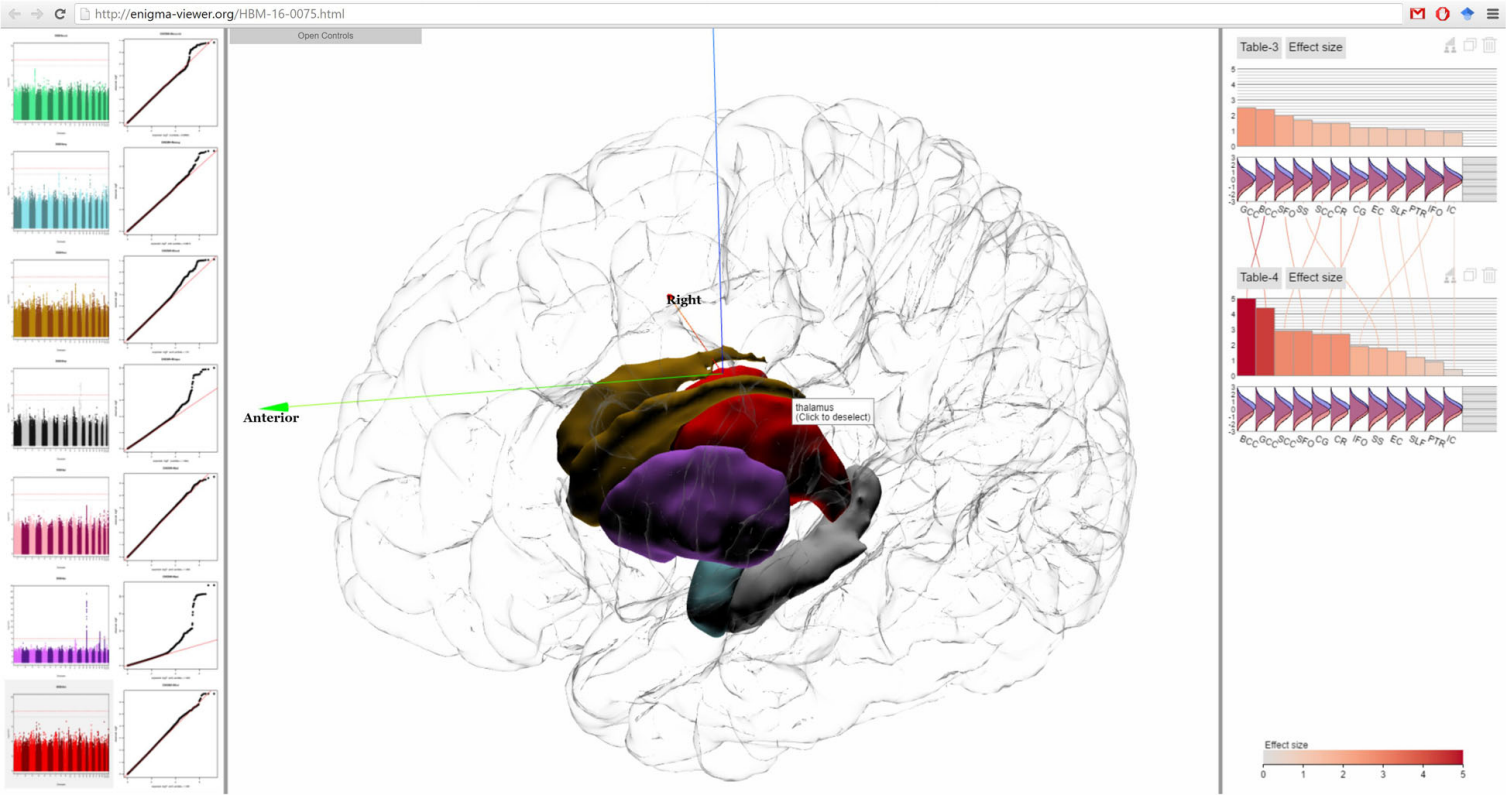
Querying Manhattan plot will highlight 3D regions. The center view displays seven sub-cortical regions and the Manhattan and QQ plots from GWAS analysis. Those regions are colored using the same colors as those in the Manhattan plots

Our tool supports a set of interaction techniques: linking and brushing, zooming, panning, and view reconfiguration. The viewer can manually select interesting brain regions under study in the effect size bar charts and examine the spatial location in the 3D view via brushing [46]. Multiple regions of interest can be selected and visualized and also linked to the artificial Manhattan plot (Fig. 5).

Our tools also support drag-and-drop operations to facilitate inter- and intra-study comparison. The bar charts can be dragged and dropped next to other bar chart or to the spatial view. Dragging-and-droping a bar chart next to other bar charts can be used to rearranged the layout of multiple bar charts, which could make comparison between different studies easier. The user can also drag the bars from the right-side bar chart to the 3D glass brain regions. This action results in the display of a region-centric comparison chart. Brain regions currently being selected will be shown. This design provides a region-specific comparison mechanism.

### Multimodality visualization

**Fig. 6:**
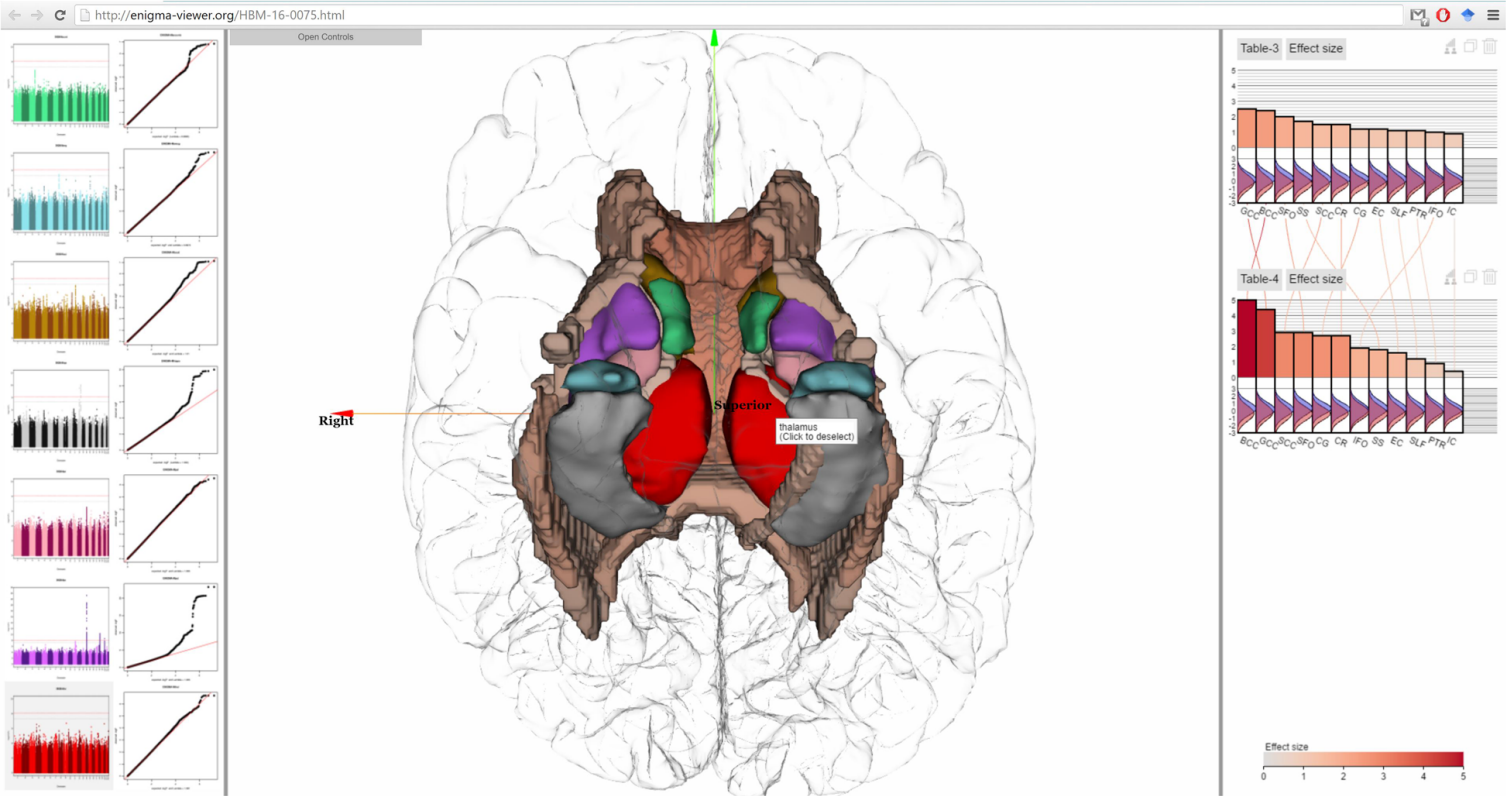
Cortical and sub-cortical regions are highlighted in the glass brain. The regions in 3D view have the same color as those used in Manhattan plots or bar charts

**Fig. 7:**
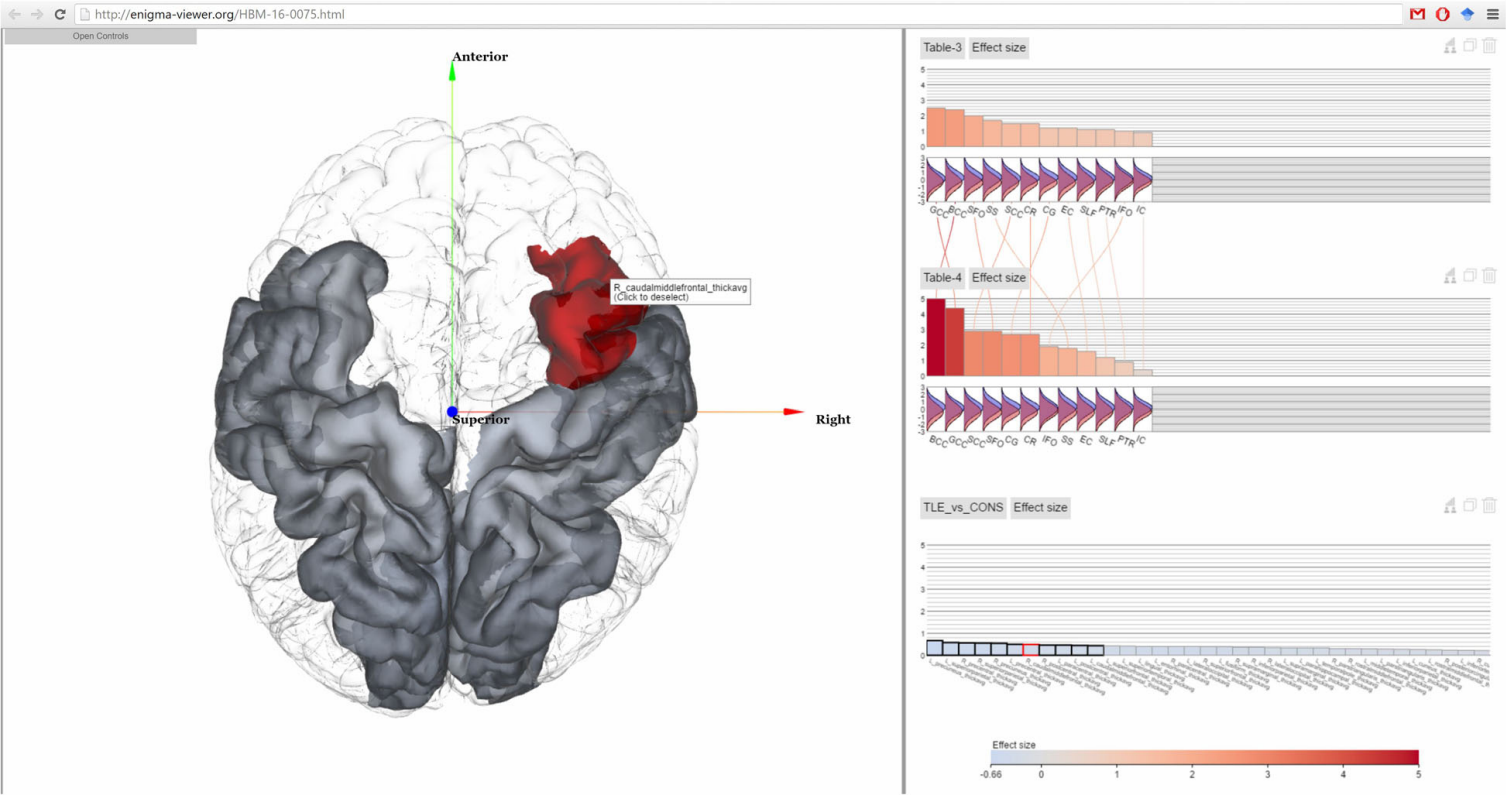
One cortical region is highlighted in the glass brain. Cortical regions can be visualized and interacted with sub-cortical regions together using the same method. The *TLE_vs_CONS* dataset is from a study on 68 cortical regions on temporal lobe epilepsy and contains 339 normal controls and 415 patients [48]

Our visualization supports multimodality visualization in that multiple attributes of brain regions can be visualized together. As can be seen from Fig. 6, the Manhattan and QQ plots are linked to the 3D brain regions. Figure 7 also shows that the plot modality and the chart modality are both linked to the 3D view. This makes it convenient to visualize multiple attributes of brain regions in the 3D view.

### Implementation

ENIGMA-Viewer is implemented in Google WebGL and JavaScript and can be executed on major web-browsers such as Safari, Firefox, and Internet Explorer, without requiring any third-party software or add-ons. No account or authorization is required to use ENIGMA-Viewer and users are encouraged to email the developers with all comments and suggestions.

The sub-cortical geometry data is extracted from sub-cortical white matter atlas using marching cubes algorithm [31]. To ensure a fast loading, the mesh is only extracted when a region is selected in the 3D view. We only stored the atlas volume to reduce the data to be loaded to the browser.

## Results

In this section we show three examples [33, 47, 48] of real world applications of this tool. The following work uses real data which are from ENIGMA group and includes both white matter and cortical gray matter comparisons.

### White matter comparisons

In Fig. 1, values from two result tables from a recent work [33] are displayed. In this study, DTI images of cohorts of schizophrenia patients (*n* =177) and controls (*n* =249) are compared to test if differences in the trajectories of white matter tract development influenced patient–control differences in FA and if specific tracts showed exacerbated decline with aging.

The top chart, named Table-3, shows the effect sizes of impact of diagnosis on white matter FA values. The bottom chart, named Table-4, shows the effect sizes of patient-control FA value decline (unit/year).

The scented lines reduce the mental cost of context switching when viewing the two bar charts from different tables. The two charts contain the same set of white matter regions but each region has different effect sizes in two charts. It can be seen from the lines connecting two charts that the rankings of effect sizes are different but BCC (Body of corpus callosum) and GCC (Genu of corpus callosum) are the two regions that show the highest patient-control difference in both charts.

The visual fusion of statistics data and 3D structure data enable users to further exam the spatial distributions of these statistics. The user can drag the tables onto the spatial view, which shows the color encoded brain white matter structures. This, alone with the in place charts, immediately reveals that the regions showing the highest effect sizes are in the middle-frontal areas, especially for data depicted in Table-4. Compare to those in Table-3, the middle-frontal areas still show the highest effect sizes, but they are not as outstanding as they are in Table-4.

**Fig. 8:**
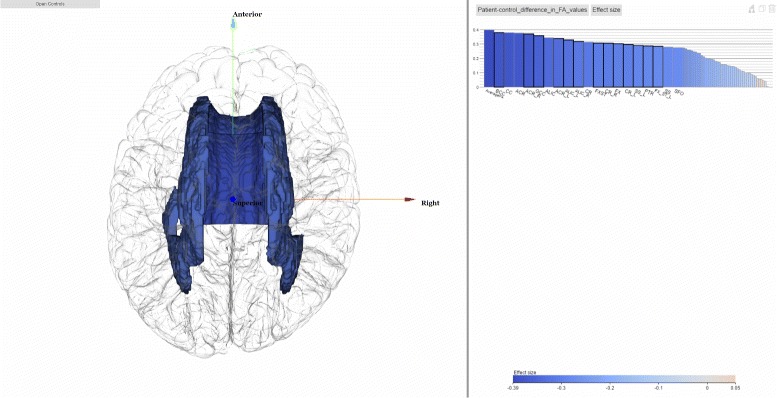
White matter comparison results from [47] are shown. Only bars with high effect sizes are left with original width and with labels shown. Bars with low effect sizes are made smaller and their labels are hidden, but they are still in the chart to provide context information

Figure 8 shows a meta analysis on brain white matter in order to identify brain regions with FA differences between schizophrenia patients and controls. The dataset comprises 30 cohorts from 14 countries totalling 2391 healthy controls and 1984 individuals with schizophrenia [47]. In the visualization, to make sure regions with more importance, i.e. higher effect sizes, are visible in the bar chart, we use focus+context method to make enough space for bars representing these regions as well as their labels. For regions with less importance, the bars can be made narrow but still in context. The focus+context design allow the brain scientists to focus on a few brain regions while keeping other regions easily accessible when needed.

### Cortical thickness comparisons

**Fig. 9:**
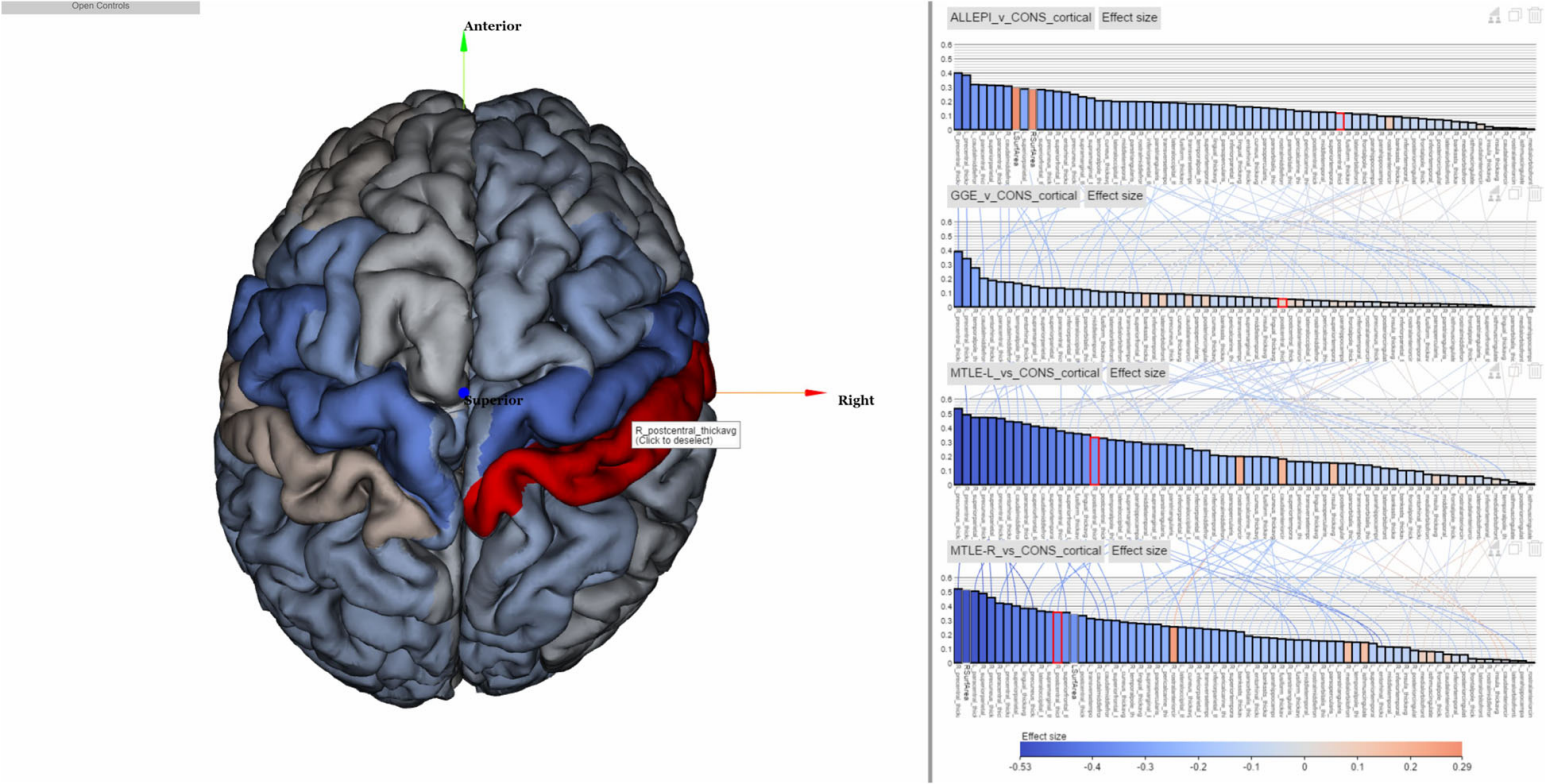
Results from [48] are shown. The four charts shows comparison results of matched healthy control group against four different groups of epilepsy in terms of cortical thickness. The symmetrical pattern can be seen in the left 3D visualization but the regions with actual highest effect sizes should be found from the chart visualization since those regions are occluded by other regions in the 3D visualization

The example in Fig. 9 shows part of the results from a study [48] which contains multiple comparisons. The study pools data from 24 research centres worldwide to identify reliable neuroimaging biomarkers in epilepsy. Here the four charts from top to bottom show comparison of gray matter (cortical thickness) between a matched healthy group (*n* =1727) and four epilepsy groups: all types of epilepsy in aggregate (ALLEPI, *n* =2149), genetic generalised epilepsies (GGE, *n* =367), mesial temporal lobe epilepsies left (MTLE-L, *n* =415) and mesial temporal lobe epilepsies left right (MTLE-R, *n* =339). These four charts contain the same 70 cortical regions but they have different effect sizes in different comparisons. From the scented lines we can see that the rankings of regional effect sizes are different among different comparisons.

The linking of statistics data and spatial structure data via highlighting enables users to see information which is otherwise difficult to notice. In Fig. 9, the color encodings of brain cortical meshes show the results from the GGE comparison (the second topmost chart). We can see from the mesh that the result shows seemingly left-right symmetrical pattern, which is difficult to observe with bar chart only representation. On the other hand, if we look at only the brain mesh visualization we may assume that the left and right poster central regions are the two most abnormal regions since they are the most reddish color compared to other regions. However when mouse hovering those two regions’ meshes, the linked bars are highlighted (with red boarder) in the bar charts and we can see that our previous assumption is not true since they are not the ones with highest positive effect sizes in this group but the right banksst (banks of the superior temporal sulcus) regions are. This is hard to observe in the 3D brain mesh visualization alone because the banksst regions are occluded by other cortical regions and even rotating the brain mesh cannot make this readily obvious. It is thus important to link both statistics data and spatial structure data in one visualization.

## Discussion

This section discusses alternative designs and the logical next steps to improve the usefulness and usability of the visualization design.

### The interpretation of effect size

Visualizing effect sizes only is not enough; neuroscientists must interpret and evaluate effect size for its practical significance and interpret other factors that cause the differences in different studies. Thus, how to interpret effect size is also a crucial question. The common practice here is to use the benchmarks for “small”, “medium”, and “large” effects. However, often this categorization depends on the domain of use and applying existing guidelines directly can be inappropriate.

Because such interpretation often depends on prior effect sizes in the related literature, both the size and nature of the effect should be included in the interpretation to increase the practical significance. Effect size also depends on multiple factors such as the context of the study, the importance of the outcomes, etc. Thus, visualizing the effect sizes and automatic searching and showing related context information to assist effect size interpretation within and between studies is the logical next step in truly helping neuroscientists’ decision-making.

### Limitations of the current study

Though we have attempted to use good visual design principles to guide our study, validation is the next step in creating truly meaningful tools for neuroscientists. One cannot assume that understanding has been gained from data represented graphically merely from the fact that the visualization has been presented; still less can one assume that a specific visual analysis can integrate all the capabilities required for multifaceted, spatially complex data analysis. Perception and cognition are complex and evaluation of visualization approaches for information presentation and interpretation is much needed to validate our design.

Our next step is also to improve data processing, handling, sharing, and collaborations using common infrastructure and data format standards. We plan to follow the data-format protocols defined by the ENIGMA consortium. We also plan to design dynamic and interactive queries among variables to support dynamic data analysis and to maximize flexibility to cross-link or brush-and-link across displays to find data relationships and compare and filter to remove redundancy. We will integrate the computational solutions and construct the entire workflow so that analysis and visualization can be integrated in a single framework, thus easing computing, data exploration, and human understanding of the massive datasets. The linked multiview visualization also provides a solution for brain scientists to understand how the statistics data provenance [49] in that the factors, such as cohort distributions, that are used to produce the results can be visualized in addition to the final statistics displayed on the 3D brain.

## Conclusions

Meta-analytic thinking would considerably facilitate knowledge accumulation in brain science. In principle, meta-analysis does not overemphasize the outcomes of statistical tests in individual studies; instead, it stresses the need to explicitly design and place studies in the context of prior research. The visualization, reporting, and interpreting of effect sizes are ways to make more explicit comparisons across cohorts in a meta-study. It is particularly beneficial to incorporate prior effect sizes to guide the findings even before a study is conducted. Visualization tools improve accessibility and facilitate quick pattern finding. Our present tool has been used by several teams and has demonstrated the power of visualization to assist reporting and interpreting effect size measures.

## Abbreviations

AFNI: Analysis of Functional NeuroImages
ALLEPI: ALL types of EPIlepsy in aggregate
BCC: Body of Corpus Callosum
DTI: Diffusion Tensor Imaging
ENIGMA: Enhancing Neuro Imaging Genetics Through Meta Analysis
FA: Fractional Anisotropy
FMRI: Functional Magnetic Resonance Imaging
FSL: FMRIB (Oxford Centre for Functional MRI of the Brain) Software Library
GCC: Genu of Corpus Callosum
GGE: Genetic Generalised Epilepsies
GWAS: Genome Wide Association Study
JHU: Johns Hopkins University
MTLE-L: Mesial Temporal Lobe Epilepsies-Left
MTLE-R: Mesial Temporal Lobe Epilepsies-Right
NITRC: Neuroimaging Informatics Tools and Resources Clearinghouse
ROI: Region of Interest
SOLAR: Sequential Oligogenic Linkage Analysis Routines
